# Role of extracellular matrix proteoglycans in immune cell recruitment

**DOI:** 10.1111/iep.12428

**Published:** 2022-01-25

**Authors:** Anna L. Gray, Nabina Pun, Amanda J. L. Ridley, Douglas P. Dyer

**Affiliations:** ^1^ Wellcome Centre for Cell‐Matrix Research Faculty of Biology, Medicine and Health Manchester Academic Health Science Centre Lydia Becker Institute of Immunology and Inflammation University of Manchester Manchester UK; ^2^ Geoffrey Jefferson Brain Research Centre Northern Care Alliance NHS Group Manchester Academic Health Science Centre University of Manchester Manchester UK

**Keywords:** adhesion, chemokine, glycosaminoglycan, leucocyte, migration, proteoglycan

## Abstract

Leucocyte recruitment is a critical component of the immune response and is central to our ability to fight infection. Paradoxically, leucocyte recruitment is also a central component of inflammatory‐based diseases such as rheumatoid arthritis, atherosclerosis and cancer. The role of the extracellular matrix, in particular proteoglycans, in this process has been largely overlooked. Proteoglycans consist of protein cores with glycosaminoglycan sugar side chains attached. Proteoglycans have been shown to bind and regulate the function of a number of proteins, for example chemokines, and also play a key structural role in the local tissue environment/niche. Whilst they have been implicated in leucocyte recruitment and inflammatory disease, their mechanistic function has yet to be fully understood, precluding therapeutic targeting. This review summarizes what is currently known about the role of proteoglycans in the different stages of leucocyte recruitment and proposes a number of areas where more research is needed. A better understanding of the mechanistic role of proteoglycans during inflammatory disease will inform the development of next‐generation therapeutics.

## 1 INTRODUCTION

Proteoglycans are extracellular matrix components that play a wide‐ranging role in the immune system and in wider biology.^1^ Proteoglycans are key regulators of immune cell (leucocyte) recruitment and positioning during the inflammatory process and associated diseases such as rheumatoid arthritis, atherosclerosis and cancer.^2^

Leucocyte recruitment is a key component of the immune response where these cells, produced largely in the bone marrow, are recruited from the circulation and into tissues as required during specific phases of the immune response.^3^, ^4^ Once leucocytes have been recruited from the vasculature, they then undergo further migration to achieve distinct positions within the tissue. This process occurs during inflammation to facilitate the removal of invasive pathogens and infected cells by the innate and adaptive immune response. Generally speaking, eosinophils, basophils, neutrophils and monocytes are recruited early in the immune response followed later by T and B cells. Leucocytes (primarily monocytes/macrophages) are also key during the resolution of inflammation and associated tissue damage.

Whilst the process of leucocyte recruitment and positioning via transendothelial migration has been well studied, the role of the extracellular matrix, in particular proteoglycans, is often overlooked. In this review, we will summarize what is known about the mechanistic and functional roles of proteoglycans in the process of leucocyte recruitment and also highlight gaps in our knowledge where further research is needed.

## 2 PROTEOGLYCAN AND GLYCOSAMINOGLYCAN STRUCTURE

**FIGURE 1:**
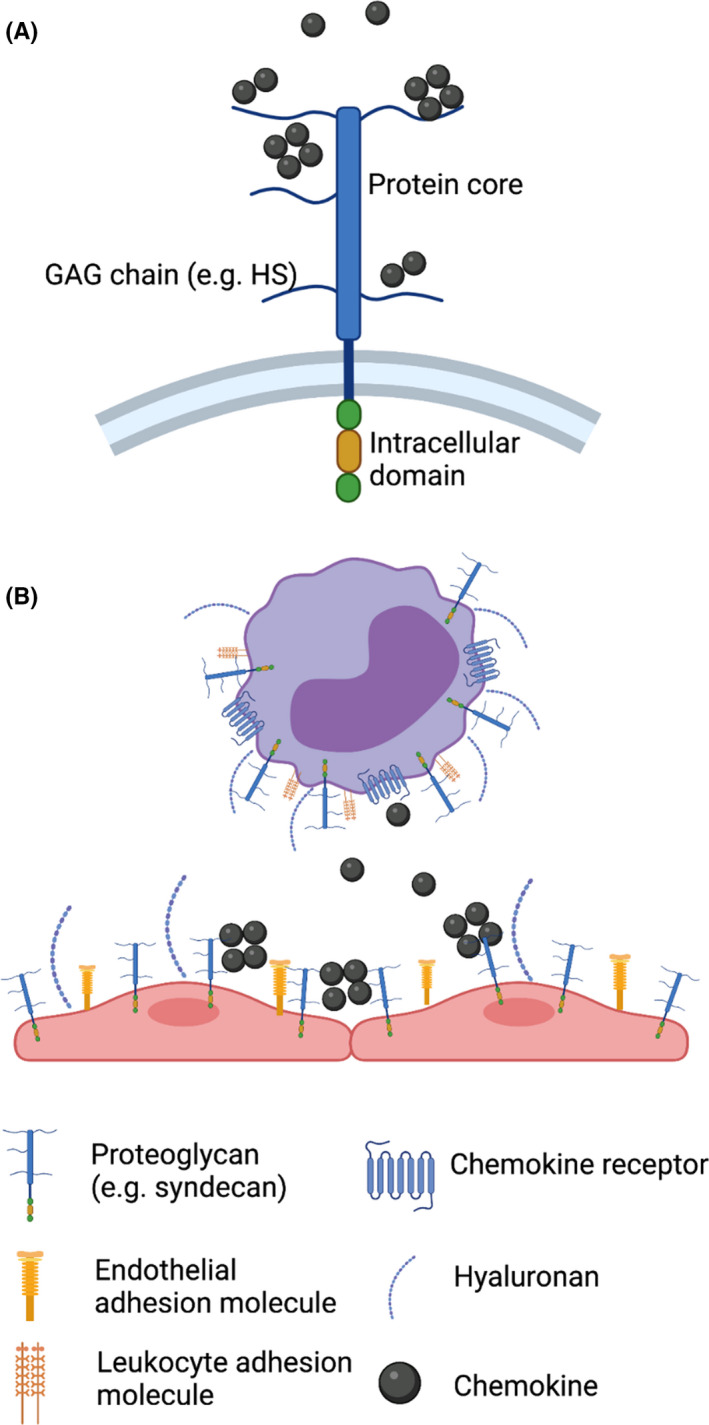
Proteoglycans regulate leucocyte recruitment. (A) Proteoglycans are composed of a protein core, depicted here embedded in a cell membrane as is the case with the syndecan family. Proteoglycans have glycosaminoglycan (GAG) side chains that can bind to a number of proteins, for example chemokines. (B) Proteoglycans on the surface of both endothelial cells and leucocytes regulate interactions between the two by masking adhesion molecules. Created with BioRender.com

Proteoglycans consist of proteins cores, either embedded in a cell membrane or soluble with sulphated glycosaminoglycan sugar side chains attached (Figure 1).^1^, ^5^ Membrane‐embedded protein cores consist of the syndecan and glypican families, whilst the soluble proteoglycans consist of serglycin, agrin, perlecan and collagen XVIII. These proteoglycan structures are found within the extracellular matrix of tissues, on the surface of the majority of mammalian cells, and are particularly prevalent on the surface of the endothelium lining blood vessels as part of the glycocalyx, and also within the basement membrane.^6^ We currently do not have much insight into the potential differential function of proteoglycans in these two distinct environments, that is luminal glycocalyx vs basement membrane.

The glycosaminoglycan (GAG) sugars that are present as side chains on the protein cores of proteoglycans are made up of repeating disaccharide units that repeat to form long unbranched (linear) chains.^7^ Both heparan sulphate (HS) and chondroitin sulphate (CS) GAGs play critical roles in leucocyte recruitment and positioning.^2^ Whilst less studied in this context, dermatan sulphate (DS) may also contribute to this process via its ability to bind to relevant proteins, for example chemokines, albeit much weaker when compared to HS.^8^ Heparan sulphate is made up of repeating units of glucuronic acid (GlcA) [which can be epimerized to iduronic acid (IdoA) during assembly] and N‐acetylglucosamine (GlcNAc).^7^ The CS chain sequence is slightly different and is made up of repeating units of glucuronic acid and N‐acetylgalactosamine (GalNAc).^9^ The DS sequence backbone is the same as CS but with some glucuronic acid residues epimerized to become iduronic acid. GAG chains are acidic and are also hydrophilic; thus, when present together they form a hydrated and ‘soft’ gel‐like structure (Figure 1B).^5^ This is particularly the case on the endothelial glycocalyx that lines blood vessels and regulates leucocyte recruitment and inflammation by the mechanisms described below.

Glycosaminoglycan chains are sulphated at various points, in the case of HS (the dominant proteoglycan GAG chain in leucocyte recruitment), N‐ and 6‐O sulphation on the glucosamine, 2‐O sulphation on the iduronic acid and more rarely 3‐O sulphation on the glucosamine.^7^, ^10^ In contrast, CS can be 2‐O‐sulphated on the glucuronic acid and 4‐O‐ and/or 6‐O‐sulphated on the N‐acetylgalactosamine residue.^9^ DS is more commonly 2‐O‐sulphated than CS, due to the epimerized iduronic acid residue. These sulphation points are critical to the ability of HS and CS GAG chains to interact with a range of different ligands, many of which are important in the leucocyte migration process (discussed below).

A range of literature has dissected the roles of GAG sulphation points in interactions with different ligands; however, there is still much to learn about the potential specificity of these interactions and the effect this may have during leucocyte recruitment in vivo. New tools to analyse the contribution of specific sulphation points to GAG interactions in a cellular context combined with increasing ability to sequence GAGs purified from biological contexts will significantly develop this field in the coming years.^11^, ^12^, ^13^

## 3 ENDOTHELIAL PROTEOGLYCANS

The vascular endothelium constitutes a monolayer of endothelial cells, which comprise the innermost cellular lining of blood vessels. These cells serve a variety of important functions, which include the coordination of inflammation and immune responses.^14^ The vascular endothelial surface is coated with a carbohydrate‐rich matrix called the glycocalyx, which in the past has been somewhat overlooked, in the context of disease. The endothelial glycocalyx is a negatively charged, membrane‐bound layer of proteoglycans and glycoproteins, which line the luminal surface of blood vessels. This protective structure was first visualized following the invention of transmission electron microscopy in the 1960s.^15^

At the luminal surface of the endothelium, syndecans and glypicans (membrane‐bound) and serglycin and agrin (soluble) are thought to be the dominant proteoglycans.^5^ The endothelial glycocalyx also contains hyaluronan, which is non‐sulphated and is anchored by the CD44 receptor, and the hyaluronic acid synthase enzyme. Some additional glycocalyx glycoproteins have a sialic acid or a fucose cap, which serve important functions in coagulation (eg, selectins, integrins and immunoglobulins) or act as endothelial adhesion molecules. Additionally, there are both endothelial‐ and plasma‐derived soluble components incorporated into this matrix.

Due to the physical properties of its components, the glycocalyx forms a thick and hydrated barrier. The intact glycocalyx is too thick to allow interaction between adhesion molecules on leucocytes and the endothelium (Figure 1B), a key component of leucocyte migration.^16^, ^17^, ^18^, ^19^ Even when projected onto endothelial protrusions, the mass of a leucocyte is insufficient to facilitate penetration of the glycocalyx.^20^

The inhibition of the first step in leucocyte recruitment by glycocalyx proteoglycans means there is missing mechanistic understanding at the heart of leucocyte recruitment that has yet to be resolved, that is how do leucocytes interact with the endothelium in the context of the glycocalyx?

## 4 HOW IS THE GLYCOCALYX REMODELLED TO FACILITATE LEUCOCYTE MIGRATION?

A number of studies have demonstrated mechanisms whereby the glycocalyx can be remodelled to facilitate leucocyte:endothelial interactions.^5^ For example, injection of the chemoattractant formylmethionyl‐leucyl‐phenylalanine (fMLP) was shown to mediate alterations in glycocalyx structure that promoted leucocyte rolling on the endothelium.^21^ Further studies demonstrated that this was by induction of heparinase leading to shedding of HS GAGs from proteoglycans facilitating access to the endothelial adhesion molecules, for example intercellular adhesion molecule 1 (ICAM‐1).^22^ More recent studies have demonstrated a similar mechanism whereby the inflammatory cytokine tumour necrosis factor (TNF) can induce heparinase‐mediated glycocalyx shedding during sepsis.^18^, ^23^ This was shown to facilitate recruitment of neutrophils and also induce side effects in the brains of sepsis patients by the GAG‐binding protein brain‐derived neurotrophic factor (BDNF).^24^

In recent years, the integral role of the glycocalyx in determining cardiovascular health and disease has been established.^25^ In chronic conditions such as rheumatoid arthritis, diabetes, sepsis, atherosclerosis and ischaemia/reperfusion injury, leucocyte recruitment can be excessive and detrimental. The presence of the glycocalyx constituents within the blood plasma can act as a biomarker for disease, since glycocalyx shedding occurs in numerous disease pathologies. Specifically shedding of syndecans (major glycocalyx components), for example syndecan‐1 by matrix metalloproteinases, into the circulation has been proposed as a marker of inflammation.^26^ Importantly, changes to the resulting composition of the glycocalyx can also drive disease through the exposure of adhesion sites, and thus the facilitation of leucocyte transmigration.

Whilst these studies, and others, have begun to develop our understanding of changes to proteoglycans during inflammation and leucocyte recruitment, a number of outstanding questions remain. For example how does the shedding of the glycocalyx fit with the studies demonstrating that proteoglycans are also essential for leucocyte recruitment (detailed later)? Presumably during inflammation, there is a balance between shedding and retention of proteoglycans that must exist to remove sufficient glycocalyx to not only allow leucocyte:endothelial interaction but also allow the pro‐migratory functions of proteoglycans.

## 5 ENDOTHELIAL PROTEOGLYCANS ACROSS VASCULAR BEDS

One issue that affects our understanding of the role of proteoglycans in leucocyte recruitment is their distribution and heterogeneity within the glycocalyx across different vascular beds, that is arterial vs venous blood vessels.

It has been suggested that glycocalyx thickness increases with vessel diameter, at least in the arterial system where the matrix is more substantial.^27^ The estimation of glycocalyx thickness is variable due to its sensitivity to processing and the differential methods used in analysis.^28^ However, it seems likely that glycocalyx morphology is different between vessel types, with the thinnest measurements recorded in capillaries and venules, ranging from 0.2 to 0.5 μm.^27^ Whereas a glycocalyx of small arteries extends 2–3 μm, a glycocalyx of larger arteries extends up to 4.5 μm. Furthermore, evidence suggests that the glycocalyx differs between the same vessel types of different organs. For instance, capillaries in the brain, heart and lung are all considered continuous with complete glycocalyx covering. However, the glycocalyx of cerebral capillaries is thicker than that of cardiac and pulmonary capillaries, likely due to its important contribution to the blood–brain barrier (BBB).^27^

Since the majority of leucocyte recruitment from the vasculature and into tissues is thought to occur within post‐capillary venules, it is possible that the glycocalyx is thinner and thus more permissive to leucocyte:endothelial interactions at this site.^3^, ^5^ It seems highly likely that the proteoglycan content and structure of the glycocalyx will differ across vascular beds and also across different tissues. Future studies are needed to specifically define proteoglycan content and structure of the glycocalyx at different vascular beds within different tissues, before and after inflammation and leucocyte recruitment, to comprehensively understand this process.

In addition to overall proteoglycan structure and content of the vascular system, the specific mechanistic role, and geographical location, of GAG sulphation is also likely to be important.^7^, ^29^ GAGs can be modified to have the sulphation points described above, and specific patterns likely mediate specific interactions within different ligands involved in leucocyte recruitment (discussed below). Whilst the sulphation of GAGs across tissues and species has been shown to be specific,^30^ we still have little information of how this varies across vascular beds and in response to different inflammatory stimuli. Thus, future studies will also need to better address the specifics in changes to GAG sulphation in defined geographical and inflammatory contexts. Such approaches now seem increasingly feasible following recent technological advances in GAG analysis.^12^, ^13^

As mentioned above, a number of studies have demonstrated that the application of factors to induce shedding of proteoglycans and their GAG chains has been shown to mediate increased rolling and migration of leucocytes from the vasculature.^5^ In contrast, other studies have shown that proteoglycans are required for leucocyte migration, where their removal actually reduces leucocyte recruitment.^31^, ^32^ There are likely a number of ways in which proteoglycans promote leucocyte recruitment.

## 6 PROTEOGLYCAN REGULATION OF CHEMOKINE FUNCTION

One of the most well‐studied functions of proteoglycans during leucocyte recruitment is their ability to interact with chemokines, whose primary function is to facilitate firm adhesion of leucocytes to the endothelium.^33^ Chemokine:proteoglycan interactions within the basement membrane are likely also key in leucocyte recruitment and trafficking. Chemokines bind to their receptors on circulating leucocytes to induce signalling events that result in integrin activation and thus firm adhesion to the endothelium. A number of years ago, it was shown that mutation of CCL2, CCL4 and CCL5, so that they could no longer bind to GAGs, ablated their ability to mediate leucocyte recruitment to the peritoneum of mice.^34^ Subsequently, a range of studies have demonstrated a similar function for other chemokines and have demonstrated a clear hierarchy in the ability of chemokines to bind to GAGs.^35^, ^36^, ^37^, ^38^, ^39^, ^40^, ^41^, ^42^, ^43^, ^44^ A number of studies have also explored the potential specificity in binding to different sulphation patterns on GAGs.^45^, ^46^ Indeed given the therapeutic potential in targeting chemokines, the chemokine:GAG interaction is an ongoing focus for potential new therapeutics to target inflammatory disease.^47^

Despite this range of research, the mechanistic importance of chemokine:GAG interactions has yet to be fully understood.^48^ It seems likely that interaction with GAGs within the luminal glycocalyx is important to retain chemokines at inflammatory sites in the presence of blood flow. However, there is very limited evidence for the common assertion that GAGs facilitate formation of chemokine gradients within the vasculature,^49^ where gradient formation has more commonly been observed within the lymphatic system or tissues in the absence of blood flow.^50^ CCL19 and CCL21 have been shown to have specific functions, by their differential interactions with HS and CS proteoglycans, in trafficking of dendritic cells within the lymphatic system.^50^, ^51^, ^52^, ^53^ Furthermore, given recent discoveries on the importance of self‐generated gradients there remains the exciting possibility that GAGs are important in this process both within tissues and the vasculature.^54^

The other mechanistic role of chemokine:GAG interactions is in protection from proteolysis,^55^, ^56^ this again seems likely to be important in chemokine‐mediated leucocyte recruitment. Given that these interactions will result in both bound and non‐bound chemokine at any given time, the cloud hypothesis has recently been proposed.^48^, ^57^ This states that GAGs mediate retention of a local cloud of soluble chemokine that is available to bind to circulating leucocytes within the vasculature to facilitate their firm adhesion.

More recently, a number of studies, including our own, have demonstrated that as well as binding to GAGs certain chemokines, for example CXCL4 and CXCL12, can remodel the structure of HS.^39^, ^44^ This involved cross‐linking of individual GAG chains to render them less mobile within a biophysical model of the cell membrane lipid bilayer. Cross‐linking also resulted in a reduced thickness of the glycocalyx‐like structures formed by HS GAG chains. Work is now ongoing in our laboratory to determine the biological function of these remodelling events; for example, can chemokines bind and alter the endothelial glycocalyx structure on blood vessels to increase its permeability and enable leucocyte recruitment in vivo?

As with the other areas of proteoglycan function in leucocyte recruitment, we are only at the beginning of our understanding of this complex biological problem. Exciting technological developments will be at the heart of future studies to understand the mechanistic function of chemokine:GAG interactions. For example why do chemokines exhibit such a wide range of affinities for GAGs, why can certain chemokines modify GAG structure and what is the role of specific GAG sulphation patterns in chemokine function?^48^

## 7 GAGs AND ADHESION MOLECULES

Numerous studies have highlighted the importance of sialyl‐Lewis X (SLe^x^) as a selectin ligand in the interactions between leucocytes and endothelial cells during leucocyte migration.^58^, ^59^, ^60^ Another direct function of proteoglycans, by their GAG side chains, in facilitating leucocyte recruitment is their interaction with leucocyte adhesion molecules, for example selectins. The ability of L (leucocyte)‐ and P (platelet)‐selectins but not E (endothelial)‐selectins, to bind GAGs, has been demonstrated.^58^ P‐selectin has also been shown to bind to CS, in a model of metastatic breast cancer, where it is involved in facilitating tumour cell adhesion to platelet and endothelial cells, promoting tumour metastasis.^59^ This interaction has been shown to play a role in selectin‐dependent cell adhesion, for example neutrophil and monocyte rolling on the endothelium.^60^, ^61^, ^62^, ^63^, ^64^

## 8 PROTEOGLYCAN:CYTOKINE INTERACTIONS

Whilst much less appreciated than specific chemokine interactions, there is a wide range of literature demonstrating that proteoglycans, via GAGs, can also bind to, and modulate the function of, cytokines more generally. Given that many of these cytokines are pro‐inflammatory, these interactions again play an important, if less direct, role in leucocyte recruitment.

Glycosaminoglycans (GAGs) have been shown to bind to interferon‐γ (IFN‐γ) with high affinity, comparable to higher affinity chemokine:GAG interactions.^65^ IFN‐γ is a cytokine that plays a key role in the complex immune response to infection, in particular by viruses, and as such plays an important role in leucocyte recruitment, for example by inducing production of the chemokines CXCL9, CXCL10 and CXCL11.^66^ The IFN‐γ:GAG interaction has been shown to reduce signalling of this cytokine through its receptor,^65^ suggesting overlapping binding sites on IFN‐γ for its receptor and GAGs. Thus, it seems likely the function of this interaction is independent of signalling, in contrast to the fibroblast growth factor (FGF) system.^65^ However, GAGs can also promote IFN‐γ‐mediated outcomes, suggesting that this interaction may facilitate the function of this cytokine through a currently undefined mechanism. Furthermore, a number of cytokines may bind to GAGs, for example IL‐2, IL‐5, IL‐6, IL‐7, IL‐12 and IL‐27, within the tissue extracellular matrix.^2^

Various members of the transforming growth factor‐β (TGF‐β) cytokine superfamily have been shown to contain heparin binding sites; for example, TGF‐β1 and TGF‐β2 are described to bind HS PGs.^67^ Although the effect of HS:TGF‐β interactions on cytokine activity has not been fully elucidated, it could be speculated that as these cytokines are comparatively small, HS binding may interfere with TGF‐β receptor signalling.^67^

The function of proteoglycan:cytokine interactions remains unclear; however, it seems likely that they are important in cytokine localization, in protection from proteolysis and in regulation of signalling through their receptors. The effects of these interactions on leucocyte recruitment are indirect in that these cytokines are involved in the inflammatory process that results eventually in recruitment of leucocytes. This again highlights that further mechanistic work is needed to understand the role of proteoglycans in regulation of cytokine function and immunology more widely.

## 9 GAGs AND TLR SIGNALLING

Toll‐like receptors (TLRs) are transmembrane receptors with a critical role in the activation of the innate immune response through recognition of pathogen‐associated molecular patterns (PAMPs) and damage‐associated molecular patterns (DAMPs).^68^ This process is key in signalling to the immune system to recruit leucocytes to sites of infection to fight pathogenic agents. HS and CS GAGs have been shown to act as DAMPs by signalling through TLR4.^69^, ^70^

Heparan sulphate can activate TLR4 on dendritic cells, in vitro, producing dendritic cell (DC) activation and alloreactive T‐cell responses.^71^ TLR4‐dependent HS signalling has also been shown to mediate recruitment of neutrophils to the pancreas.^72^ Given the presence of HS GAGs on the endothelial surface and within the tissue extracellular matrix, it would make sense that their shedding during disease would be an important signal to the immune system that pathogens are present within the vasculature and surrounding tissues. There are still a number of questions around the role of proteoglycans as DAMPs and in facilitating leucocyte recruitment that can now be explored using the advancing tools in the area.

## 10 LEUCOCYTE PROTEOGLYCANS

Whilst the majority of the research understanding the role of proteoglycans in leucocyte recruitment is within the context of the endothelial luminal glycocalyx, we are now beginning to understand the function of proteoglycans on leucocytes themselves. A number of studies have demonstrated the presence of proteoglycans, either directly or indirectly, on the surface of neutrophils, monocytes, macrophages, mast cells and T cells. Indeed, surface proteoglycans are required for entry of viruses into cells such as leucocytes, including SARS‐CoV‐2.^73^, ^74^, ^75^

The proteoglycan Syndecan‐1 has been found to be expressed at higher levels on leucocytes during inflammation and disease. For instance, neutrophils and plasma cells from patients with type 2 diabetes or systemic lupus erythematosus (SLE) have been shown to enhance their expression of syndecan‐1, relative to healthy controls.^76^, ^77^ Genetic ablation of the proteoglycan syndecan‐1 in monocytes and neutrophils reduces their ability to adhere to endothelial cells in vitro.^78^

An early study demonstrated that acidic mucopolysaccharides, resembling chondroitin sulphate, could be isolated from human leucocytes.^79^ Further studies have then gone on to report that human leucocytes can indeed synthesize and secrete glycosaminoglycans,^80^ with chondroitin 4‐sulphate being thought to represent the major component.^81^ Furthermore, there is indirect evidence for proteoglycans on the surface of T cells; the entry of human T‐cell leukaemia virus (HTLV) into CD4^+^ T cells^74^ or herpesvirus 8 (HHV‐8) into B cells^73^ all requires heparan sulphate.

Serglycin has been demonstrated to facilitate storage granule formation in mast cells^82^ and T cells.^83^ Proteoglycans have also been detected on B cells where they go through structural changes during development and play a role in the survival of long‐lived plasma cells.^84^, ^85^, ^86^ Eosinophils have been shown to have cell surface proteoglycans that change in response to cytokine stimulation.^87^

CXCL8, which can bind to GAGs, has been shown to bind to the surface of neutrophils, where GAG‐mediated inhibition of this interaction reduced in vitro chemotaxis of neutrophils and reactive oxygen species (ROS) production.^88^ Additionally, enzymatic removal of GAGs has also been shown to ablate migration of neutrophils, in vitro.^31^ Whilst the mechanism underlying these observations is unclear, the authors propose that interactions between the chemokines CXCL8 and proteoglycans on the leucocyte surface promotes locomotion by creating local stores of ligand. This mechanism would provide a currently overlooked understanding of chemokine function in addition to binding to proteoglycans on the endothelial surface.

Indirect evidence from a number of papers has suggested that proteoglycans are on the surface of monocytes and may be functional. The GAG‐binding chemokines CXCL4 and CCL5 can both bind to the monocyte surface.^89^

The wider presence and function of proteoglycans on the leucocyte surface is an exciting research topic with huge potential for a better understanding of leucocyte recruitment and potential therapeutic targeting of inflammatory‐based disease. It seems highly likely that proteoglycans on the leucocyte surface have a key role in fighting infection, given that the glycocalyx represents the first part of a cell that any intracellular pathogens encounter. The proteoglycans, within the glycocalyx, play an important role during inflammation and represent a potential therapeutic target during disease.

## 11 CONCLUSION

Together, these studies emphasize that proteoglycans are key modulators of leucocyte recruitment and positioning, as well as the wider immune response. However, this review also highlights that much more research is needed to improve our current understanding regarding the architecture, expression patterns and functional role of proteoglycans in inflammation and disease. Greater understanding will facilitate better targeted therapeutic interventions, such as GAG mimetics, in inflammatory diseases such as rheumatoid arthritis. We are now at an exciting time in this field where development of new technologies, particularly GAG analytics, will facilitate new and exciting discoveries in the near future.
